# AC/DC Fields Demodulation Methods of Resonant Electric Field Microsensor

**DOI:** 10.3390/mi11050511

**Published:** 2020-05-19

**Authors:** Pengfei Yang, Xiaolong Wen, Zhaozhi Chu, Xiaoming Ni, Chunrong Peng

**Affiliations:** 1 School of Applied Science, Beijing Information Science and Technology University, Beijing 100192, China; pfy@bistu.edu.cn (P.Y.); nxm723@bistu.edu.cn (X.N.); 2 School of Mathematics and Physics, University of Science and Technology Beijing, Beijing 100083, China; xiaolongwen@ustb.edu.cn; 3 Institute of Microelectronics of Chinese Academy of Sciences, Beijing 100029, China; chuzhaozhi@ime.ac.cn; 4 State Key Laboratory of Transducer Technology, Aerospace Information Research Institute, Chinese Academy of Sciences, Beijing 100094, China

**Keywords:** electric field sensor, resonance, micro-electro-mechanical systems (MEMS), AC/DC electric fields, demodulation methods, frequency bandwidth, power systems

## Abstract

Electric field microsensors have the advantages of a small size, a low power consumption, of avoiding wear, and of measuring both direct-current (DC) and alternating-current (AC) fields, which are especially suited to applications in power systems. However, previous reports were chiefly concerned with proposing new structures or improving the resolution, and there are no systematic studies on the signal characteristics of the microsensor output and the demodulation methods under different electric fields. In this paper, the use of an improved resonant microsensor with coplanar electrodes, and the signal characteristics under a DC field, power frequency field, and AC/DC hybrid fields were thoroughly analyzed respectively, and matching demodulation methods derived from synchronous detection were proposed. We theoretically obtained that the frequencies of the detectable electric fields should be less than half of the resonant frequency of the microsensor, and that the sensitivities of the microsensor were identical for AC/DC hybrid fields with different frequencies. Experiments were conducted to verify the proposed demodulation methods. Within electric field ranges of 0–667 kV/m, the uncertainties were 2.4% and 1.5% for the most common DC and 50 Hz power frequency fields, respectively. The frequency characteristic test results of the microsensor were in agreement with those of the theoretical analysis in the range of 0–1 kHz.

## 1. Introduction

Measurement of electric fields in the vicinity of high-voltage equipment in a power system is essential for various applications, including ensuring personal and equipment safety in live-line maintenance [1], identifying faulty insulators [2,3], non-contact voltage measurements [4,5], avoidance of power lines from helicopters [6], ice accretion detection on power lines and electromagnetic environment assessment [7,8], etc.

Recently, high-voltage, direct-current (HVDC) power transmission lines have been developed rapidly due to the advantages of a long transmission distance, large transport capacity and low cost [9,10]; additionally, hybrid alternating-current (AC) and direct-current (DC) lines have been erected because of the scarcity of the power transmission line corridor area [8], which requires the measurement or monitoring not only of the DC electric fields, but also of the AC/DC hybrid fields. Thus, in addition to detecting the power frequency electric fields and their harmonics, it is also necessary to detect the DC fields and AC/DC hybrid fields in the power systems.

A variety of electric field sensors have been reported for power system applications [11,12,13,14,15]. The most commonly-used devices for AC field measurements are electro-optical sensors (EOSs). However, EOSs are applicable to pulse or AC rather than DC field measurements and suffer from an intrinsic temperature instability due to the pyroelectric effect and thermal expansion of the material [16]. DC field measurements are known to be comparatively more complicated than AC field measurements due to the lack of periodic change with respect to time. The conventional equipment for measuring DC fields is field mills, which convert the DC field into an alternating field by employing an electrically grounded shutter to periodically shield sensing electrodes from the incident DC field. However, a high power consumption, motor wear and bulky volume make them unsuitable for long-term measurement and large-scale networking [17].

Due to small size, low power consumption, wear avoidance, and an ability to measure both DC and AC electric fields, electric field sensors based on micro-electro-mechanical systems (MEMS) technology are particularly suitable for applications in power systems [6,16,17,18,19,20,21,22,23,24,25,26,27,28,29,30,31,32]. At present, most of the reported electric field microsensors (EFMs) are based on the charge induction principle similar to the field mills. There are also a variety of alternative approaches, such as the effect of electrostatic force and steering electrons [6,16,17,27]. The reported EFMs are designed to work at their mechanical resonant frequencies for high sensitivities. So far, the existing reports concerning resonant EFMs mainly concentrate on the sensors themselves, and verify their performance by measuring DC electric fields. There are only a few reports on AC electric field detection of the resonant EFMs. In 2009, Wijeweera et al. demonstrated the first 60 Hz AC electric field measurements of resonant EFM using a lock-in amplifier with a time constant of 3 ms [1]. In 2011, the author reported 50 Hz AC field tests based on a synchronous demodulation method [26]. In 2016, employing a lock-in amplifier, Chen et al. presented the 610 Hz AC field detection of resonant EFM [27]. Nevertheless, the output signal characteristics, the demodulation methods and the experimental verification of the resonant EFMs under different types of electric fields, especially for AC/DC hybrid fields, have not been systematically studied, which is crucial for the product development and the application of the EFMs in power systems.

In this paper, we have carried out comprehensive studies on the output signal characteristics, the demodulation methods and experimental verification based on the improved resonant EFM with coplanar electrodes under a DC field, power frequency field and AC/DC hybrid fields. The output signal characteristics and the demodulation methods based on synchronous detection are analyzed theoretically. Meanwhile, the EFM bandwidth of detectable fields is explored. Furthermore, experimental verification is performed.

## 2. Resonant EFM with Coplanar Electrodes

### 2.1. Operating Principle

**Figure 1:**
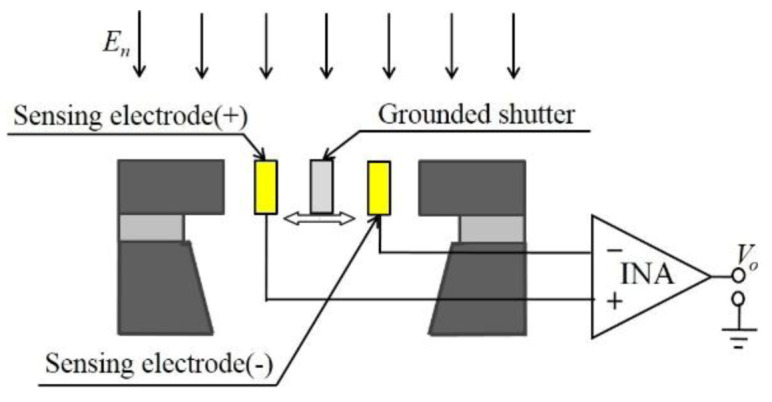
Operating principle of the resonant electric field microsensor (EFM) with coplanar electrodes.

**Figure 2:**
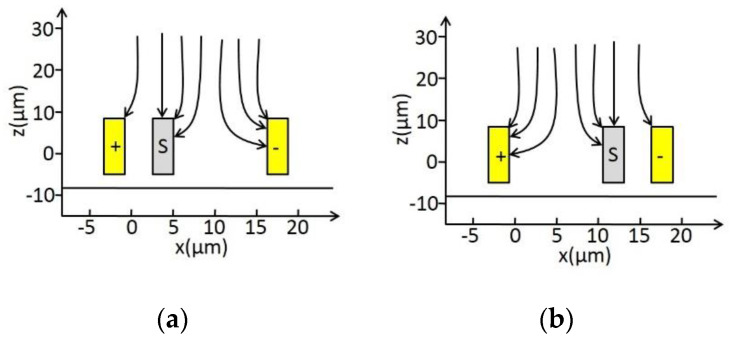
Electric field line distribution of the shutter (s) at different positions: (**a**) Proximity to sensing electrode (+); (**b**) Proximity to sensing electrode (−).

The operational principle of the EFM is shown in Figure 1 [26]. The shutter and differential sensing electrodes are designed in the same structural layer, and thus the sidewall capacitances form the useful capacitances of the structure, which improve the electric field coupling effect. The grounded shutter travels from side to side within a gap under an incident electric field *E_n_*. As illustrated in Figure 2, when the shutter is in its leftmost position, a larger fraction of the electric field lines terminate on the negative sensing electrode than on the positive sensing electrode, and thus the electric field induces more charge on the negative sensing electrode than on the positive one. When the shutter moves to its rightmost position, the situation is reversed. Consequently, as the grounded shutter swings back and forth, it covers the sidewalls of either the positive or negative sensing electrode; then, a differential AC current is generated on the sensing electrodes. The output AC current *i_s_* is calculated according to:

(1) $$i_{s}=\varepsilon_{0}\frac{dE_{n}A}{dt}$$

where *ε_0_* is the permittivity of free space, *E_n_* is the component of the electric field normal to the sensing electrodes, and *A* is the effective area of the sensing electrodes. The current *i_s_* is converted to a voltage *V_o_* by an instrumentation amplifier (INA), and the *V_o_* is then amplified and detected.

### 2.2. Structure and Fabrication

**Figure 3:**
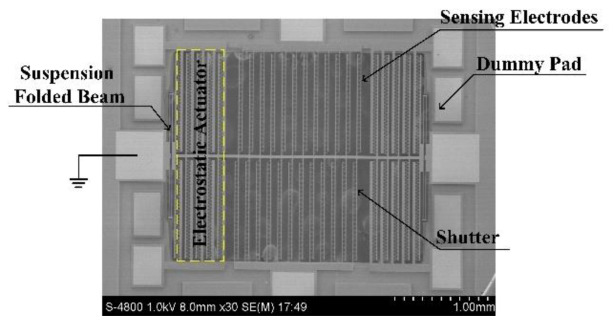
Scanning electron micrograph (SEM) photo of the fabricated EFM.

A scanning electron micrograph (SEM) photo of the EFM is shown in Figure 3. The microsensor is constructed from a resonator that is laterally actuated by electrostatic comb drives. Comb-shaped electrodes are selected as shutter and sensing electrodes to obtain a higher conversion gain than strip electrodes [26]. Both differential comb drives and differential senses are designed for feedthrough cancellation. Additionally, the improved structure design is performed. Four dummy pads are placed, separately, in the vicinity of the drive pads to further suppress the feedthrough by applying proportionally opposite driving voltages [33].

The EFMs were fabricated using a commercial SOIMUMPS process, which is a simple 4-mask level silicon on insulator (SOI) patterning and etching process [34]. The through etching of the substrate beneath the suspended silicon structures significantly reduces the air damping and yields high mechanical quality factors, and it eliminates the effects of electrostatic levitation of the vibrating structure. The microsensor size was optimized to 5 mm × 5 mm.

### 2.3. Differential Induced Charge and Output Signal

A common DC bias voltage *V_d_* and antisymmetric sinusoidal voltages *V_a_*sin(*ω_s_t*) are applied on both sides of the electrostatic actuators to drive the shutter. The microsensor was designed to work at the resonant frequency *ω_s_*. When the driving voltages *V_d_* ± *V_a_*sin(*ω_s_t*) are applied on the microsensor, the differential induced charge on the sensing electrodes can be written as [32]:

(2) $$Q\left(t\right)=Q_{A}\sin\left(\omega_{s}t+\theta \right)+Q_{0}=\left[k_{q}X_{r}\sin\left(\omega_{s}t+\theta \right)+D_{0}\right]E_{n}$$

where *Q_A_* denotes the magnitude of the differential induced charge, *θ* is the phase difference between the driving signal and the vibration displacement of the shutter, *Q_0_* is the residual charge caused by the incomplete symmetry of the sensing structure due to fabrication error, *D_0_* is the residual charge per kVm^−1^, *k_q_* represents the conversion coefficient of the vibration-amplitude-to-charge-variation per kVm^−1^, and *X_r_* is the resonant vibration amplitude of the shutter.

The output voltage of the microsensor via the INA with a gain *R_f_* is expressed as:

(3) $$V_{0}\left(t\right)=\frac{dQ\left(t\right)}{dt}R_{f}$$

## 3. Demodulation Methods

### 3.1. DC Electric Field Demodulation

Under the DC electric field, from Equations (2) and (3), the output voltage of the EFM is expressed as:

(4) $$V_{0}\left(t\right)=k_{q}X_{r}\omega_{s}R_{f}E_{n}\cos\left(\omega_{s}t+\theta \right)$$

**Figure 4:**
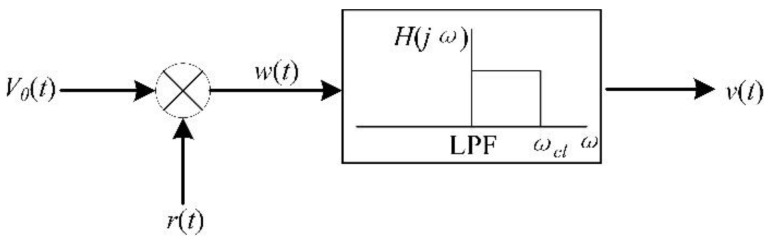
Block diagram of a synchronous demodulator.

The output voltage of the microsensor is difficult to detect directly by peak extraction due to the influence of strong background noise and the AC drive-signal feedthrough. From Equation (2), the operational principle of the resonant EFM could be interpreted as an ideal “electric field amplitude modulator”. Thus, the synchronous demodulator scheme of Figure 4 was proposed to reconstruct the measured DC field. The synchronous demodulator is based on the multiplication of the modulated signal by the AC drive signal, and the result of the multiplication is filtered by a low-pass filter.

Multiplying the output signal *V_0_*(*t*) by the AC drive signal *r*(*t*) = *V_a_*sin(*ω_s_t*) results in:

(5) $$w\left(t\right)=V_{0}\left(t\right)\cdot r\left(t\right)=k_{q}X_{r}\omega_{s}R_{f}V_{a}E_{n}\left[-\frac{1}{2}\sin\theta +\frac{1}{2}\left(2\omega_{s}t+\theta \right)\right]$$

To obtain the DC component of *w*(*t*) and eliminate noise interference, the cut-off frequency *ω_cl_* of the low-pass filter shown in Figure 4 must be much lower than the frequency *ω_s_* of the drive signal. Therefore, after the low-pass filter, the result of the synchronous demodulation is given by:

(6) $$v\left(t\right)=-\frac{1}{2}k_{q}X_{r}\omega_{s}R_{f}V_{a}\sin\theta \cdot E_{n}$$

where *k_q_*, *X_r_*, *ω_s_*, *R_f_*, *V_a_*, and *θ* are definite values. According to the second-order vibration characteristics of the resonant EFM, ideally, the value of *θ* is π/2 [35]. Equation (6) illustrates that the result of the synchronous demodulation *v*(*t*) is linear to the measured DC field *E_n_*. Therefore, the magnitude and the polarity of the measured DC electric field can be obtained by measuring *v*(*t*).

### 3.2. Power Frequency Electric Field Demodulation

If the measured field is a power frequency electric field, it can be expressed as:

(7) $$E_{n}=E_{0}\sin\left(\omega_{e}t+\varphi \right)$$

where *E*_0_ and *ω_e_* are the amplitude and frequency of the power frequency field, respectively. *φ* is the initial phase. From Equations (2), (3), and (7), the output voltage of the microsensor under the power frequency field is obtained as:

(8) $$V_{0}\left(t\right)=M\sin\left(\omega_{s}t+\theta \right)+N\cos\left(\omega_{s}t+\theta \right)+P$$

where $\left\{ \begin{array}{l} M=k_{q}X_{r}E_{0}R_{f}\omega_{e}\cos\left(\omega_{e}t+\varphi \right)\\ N=k_{q}X_{r}E_{0}R_{f}\omega_{s}\sin\left(\omega_{e}t+\varphi \right)\\ P=D_{0}E_{0}R_{f}\omega_{e}\cos\left(\omega_{e}t+\varphi \right) \end{array}\right.$.

**Figure 5:**
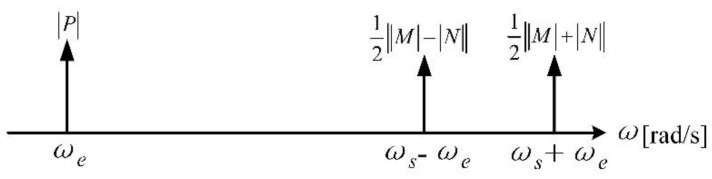
Spectrum of the microsensor output signal under the power frequency field.

Equation (8) shows that the microsensor modulates the frequency of the measured field from *ω_e_* to *ω_s_* ± *ω_e_*. However, the frequencies of the sensor output signal include not only *ω_s_* ± *ω_e_*, but also the frequency *ω_e_* of the measured field, which is directly fed through due to the structural asymmetry. Although directly extracting the signal with the frequency *ω_e_* can realize the detection of the power frequency field, this method is easily affected by co-channel interference noise and its harmonic components, and also does not reflect the advantages of the resonant EFM modulation. Hence, in order to improve the signal detection accuracy, we focus on demodulating the signal with the frequencies *ω_s_* ± *ω_e_* in the subsequent signal processing. The spectrum of Equation (8) is represented in Figure 5.

**Figure 6:**
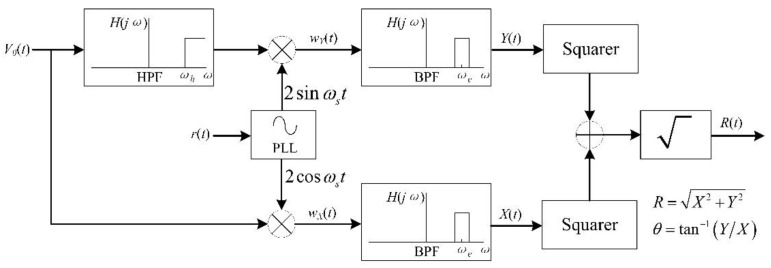
Block diagram of the high-pass-orthogonal-correlation-band-pass detection method.

Although the synchronous demodulation can also be used to reconstruct the power frequency electric field, it may lead to the weakening of the output signal strength. The reason for this is that the phase difference *θ* is often not the ideal π/2. For the DC field, we not only obtain its magnitude, but also its polarity, so the synchronous demodulation is the most appropriate method. However, for the power frequency field, we only need to get its amplitude. Hence, a novel power frequency field demodulation method of the resonant EFM based on orthogonal correlation detection technology was proposed, which was called the high-pass-orthogonal-correlation-band-pass detection method (HOCBM), as shown in Figure 6. Since we will focus on demodulating the signal with the frequencies *ω_s_* ± *ω_e_*, the sensor output first passes a high-pass filter with the cut-off frequency *ω_h_* to eliminate the feedthrough signal at the frequency *ω_e_*, *ω_h_* ∈ (*ω_e_*, *ω_s_*−*ω_e_*). Then, the orthogonal correlation is performed.

After the orthogonal correlation, *w_Y_*(*t*) and *w_X_*(*t*) can be derived as:

(9) $$\left\{ \begin{array}{l} w_{Y}\left(t\right)=M\left[\cos\theta -\cos\left(2\omega_{s}t+\theta \right)\right]+N\left[\sin\left(2\omega_{s}t+\theta \right)-\sin\theta \right]\\ w_{X}\left(t\right)=M\left[\sin\left(2\omega_{s}t+\theta \right)+\sin\theta \right]+N\left[\cos\left(2\omega_{s}t+\theta \right)+\cos\theta \right] \end{array}\right.$$

*w_Y_*(*t*) and *w_X_*(*t*) contain three frequency components: *ω_e_*, 2*ω_s_* − *ω_e_*, and 2*ω_s_* + *ω_e_*, respectively. Because we only pay attention to the power frequency field signal with the frequency *ω_e_*, band-pass filters with the center frequency *ω_e_* are selected after the orthogonal correlation, which can also prevent the interference caused by the AC drive-signal feedthrough of the microsensor. The outputs after the band-pass filter can be expressed as:

(10) $$\left\{ \begin{array}{l} Y\left(t\right)=M\cos\theta -N\sin\theta \\ X\left(t\right)=M\sin\theta +N\cos\theta \end{array}\right.$$

Expand the expression of *X*(*t*):

(11) $$X\left(t\right)=\frac{1}{2}k_{q}X_{r}E_{0}R_{f}\left[\left(\omega_{s}+\omega_{e}\right)\sin\left(\omega_{e}t+\varphi +\theta \right)+\left(\omega_{s}-\omega_{e}\right)\sin\left(\omega_{e}t+\varphi -\theta \right)\right]$$

**Figure 7:**
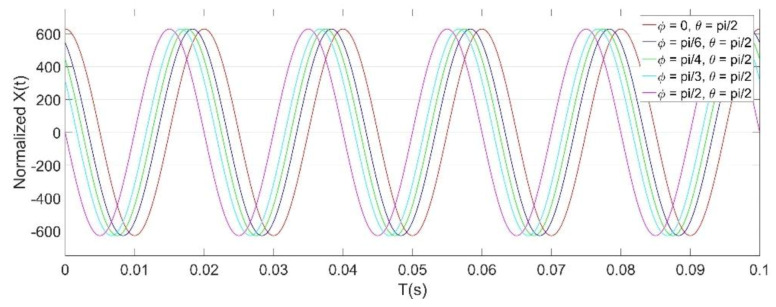
Normalized simulation results of *X*(*t*) under different initial phase *φ* values, *ω_e_* = 2π·50 rad/s, *ω_s_* = 2π·3050 rad/s.

The modulus of *X*(*t*) is linearly related to the amplitude of the power frequency field *E_0_*. Figure 7 shows the normalized simulation results of *X*(*t*) under the different initial phase *φ*, where the frequency of the power frequency field is 50 Hz, and the resonant frequency of the EFM is 3050 Hz. The simulation results show that *X*(*t*) is a sine wave with a frequency of 50 Hz.

The *R*(*t*) can be given by:

(12) $$R\left(t\right)=\sqrt{X{\left(t\right)}^{2}+Y{\left(t\right)}^{2}}=\sqrt{M^{2}+N^{2}}=\left|k_{q}X_{r}E_{0}R_{f}\right|\sqrt{\omega_{e}^{2}+\left(\omega_{s}^{2}-\omega_{e}^{2}\right)\sin^{2}\left(\omega_{e}t+\varphi \right)}$$

**Figure 8:**
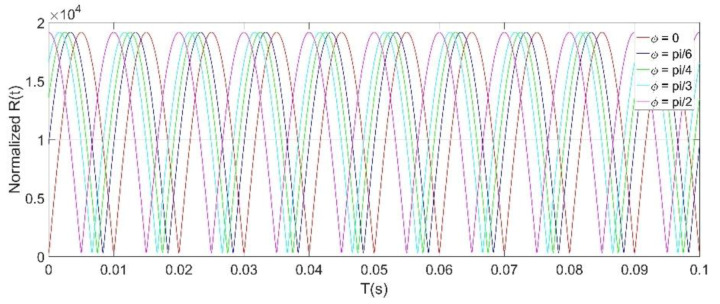
Normalized simulation results of *R*(*t*) under different initial phase *φ* values, *ω_e_* = 2π·50 rad/s, *ω_s_* = 2π·3050 rad/s.

The normalized simulation results of *R*(*t*) under different initial phase *φ* values is shown in Figure 8, which clearly illustrates that the frequency of *R*(*t*) is twice the frequency of the power frequency field. Equation (12) shows that *R*(*t*) has a linear relationship with the amplitude of the power frequency field *E_0_*. For the power frequency field detection, since *ω_e_* is much smaller than *ω_s_*, the maximum value of *R*(*t*) can be expressed as:

(13) $$R_{\max}\left(t\right)=\left|k_{q}X_{r}\omega_{s}R_{f}\right|\cdot E_{0}$$

Thus, the peak extraction method is finally used to obtain the amplitude of the measured power frequency electric field. The advantages of using the HOCBM to demodulate the power frequency field are the following:

- Because the microsensor output first passes a high-pass filter, the influence of power frequency interference and its harmonic components on the electric field measurements is suppressed.
- Unlike the synchronous demodulation of the DC electric field using a low-pass filter, band-pass filters are used to obtain the power frequency electric field signal, which inhibits the impact of the AC drive-signal feedthrough of the EFM.
- Unlike the synchronous demodulation of the DC electric field, using the HOCBM to finally extract the peak value of *R* (*t*) can avoid the change of the microsensor sensitivity caused by the *θ* variation.

### 3.3. AC/DC Hybrid Electric Fields Demodulation

If the measured fields are AC/DC hybrid electric fields, they can be expressed as:

(14) $$E_{n}=E_{d}+\sum_{i=1}^{k}E_{mi}\sin\left(\omega_{i}t+\varphi_{i}\right)$$

where *E_d_* is a DC field, *E_mi_* is the amplitude of the i-th AC field with the frequency *ω_i_*, and *φ_i_* is the initial phase of the i-th AC field. From Equations (2), (3), and (14), the output voltages of the microsensor under the AC/DC hybrid fields are obtained as:

(15) $$V_{0}\left(t\right)=N_{0}\cos\left(\omega_{s}t+\theta \right)+\sum_{i=1}^{k}\left[M_{i}\sin\left(\omega_{s}t+\theta \right)+N_{i}\cos\left(\omega_{s}t+\theta \right)+P_{i}\right]$$

where $\left\{ \begin{array}{l} N_{0}=k_{q}X_{r}E_{d}R_{f}\omega_{s}\\ M_{i}=k_{q}X_{r}E_{mi}R_{f}\omega_{i}\cos\left(\omega_{i}t+\varphi_{i}\right)\\ N_{i}=k_{q}X_{r}E_{mi}R_{f}\omega_{s}\sin\left(\omega_{i}t+\varphi_{i}\right)\\ P_{i}=D_{0}E_{mi}R_{f}\omega_{i}\cos\left(\omega_{i}t+\varphi_{i}\right) \end{array}\right.$ (i=1,2⋯k).

For the AC/DC hybrid fields, a high-pass-orthogonal-correlation-low-pass-band-pass detection method (HOCLBM) was proposed to obtain the electric field amplitude of each frequency component. There are two main differences from the demodulation of the power frequency field: (a) In order to extract all frequency components of the AC/DC hybrid fields, a low-pass filter with the cut-off frequency *ω_L_* is added between the orthogonal correlation and the band-pass filter, *ω_L_* ∈ (max{*ω_i_*}, *ω_s_*); (b) When demodulating the DC field, the band-pass filters are replaced by low-pass filters with a very low cut-off frequency. Due to the cut-off frequency of the high-pass filter, *ω_h_* ∈ (*ω_i_*, *ω_s_* − *ω_i_*), which illustrates *ω_i_* ∈ (0, *ω_s_*/2)(*i* = 1, 2,…, *k*). Therefore, in theory, the frequency range of the AC/DC hybrid fields that can be demodulated by the HOCLBM is [0, *ω_s_*/2). The output voltage *V_0_*(*t*) of the microsensor under the AC/DC hybrid fields is demodulated when adopting the HOCLBM, and the two outputs *Y_L_*(*t*) and *X_L_*(*t*) of the added low-pass filters are expressed as:

(16) $$\left\{ \begin{array}{l} Y_{L}\left(t\right)=-N_{0}\sin\theta +\sum_{i=1}^{k}\left(M_{i}\cos\theta -N_{i}\sin\theta \right)\\ X_{L}\left(t\right)=N_{0}\cos\theta +\sum_{i=1}^{k}\left(M_{i}\sin\theta +N_{i}\cos\theta \right) \end{array}\right.$$

*Y_L_*(*t*) and *X_L_*(*t*) contain all frequency components of the measured hybrid fields. After band-pass filters with the center frequency *ω_i_* or low-pass filters with a very low cut-off frequency, the *R_i_*(*t*) can be derived as:

(17) $$R_{i}\left(t\right)=\sqrt{X_{i}^{2}\left(t\right)+Y_{i}^{2}\left(t\right)}=\sqrt{M_{i}^{2}+N_{i}^{2}}\;=\left\{ \begin{array}{ll} \left|k_{q}X_{r}R_{f}\omega_{s}\right|\cdot E_{d} & \mathrm{DC}\;\mathrm{field}\\ \left|k_{q}X_{r}E_{mi}R_{f}\right|\sqrt{\omega_{i}^{2}+\left(\omega_{s}^{2}-\omega_{i}^{2}\right)\sin^{2}\left(\omega_{i}t+\varphi_{i}\right)} & \mathrm{AC}\;\mathrm{field} \end{array}\right.\;\left(i=1,2\cdots k\right)$$

By reason of *ω_i_* ∈ (0, *ω_s_*/2), we can obtain:

(18) $$\left|k_{q}X_{r}E_{mi}R_{f}\right|\sqrt{\omega_{i}^{2}+\left(\omega_{s}^{2}-\omega_{i}^{2}\right)\sin^{2}\left(\omega_{i}t+\varphi_{i}\right)}\leq \left|k_{q}X_{r}R_{f}\omega_{s}\right|\cdot E_{mi}$$

**Figure 9:**
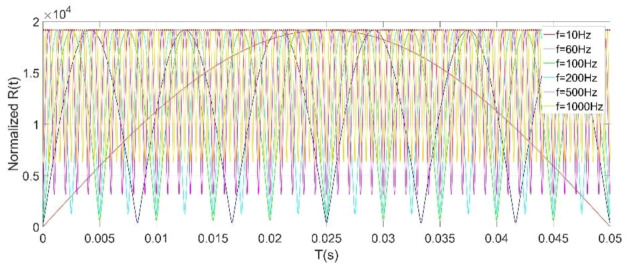
Normalized simulation results of *R_i_*(*t*) at different measured field frequencies, *ω_s_* = 2π·3050 rad/s, *φ_i_* = 0.

The peak extraction method is also eventually used to obtain the amplitude of any frequency component of the measured hybrid electric fields. From Equations (17) and (18), it can be known that the sensitivity of the microsensor by using the HOCLBM for any frequency component of the AC/DC hybrid fields in the measurable frequency range is a fixed value |*k_q_X_r_R_f_ω_s_*|. This conclusion indicates that the usage of the resonant EFMs and the HOCLBM to detect low-frequency electric fields has great advantages and is very convenient in power systems. Unfortunately, the HOCLBM cannot identify the polarity of the DC field. The normalized simulation results of *R_i_*(*t*) at different measured field frequencies are shown in Figure 9, illustrating that the frequencies of the *R_i_*(*t*) outputs are twice the frequencies of the measured AC fields, and that the maximum values of *R_i_*(*t*) for the measured AC fields at different frequencies are the same, which is consistent with the result obtained by Equation (18).

## 4. Experimental Results and Discussion

### 4.1. AC/DC Electric Fields Demodulation Verification Test System

**Figure 10:**
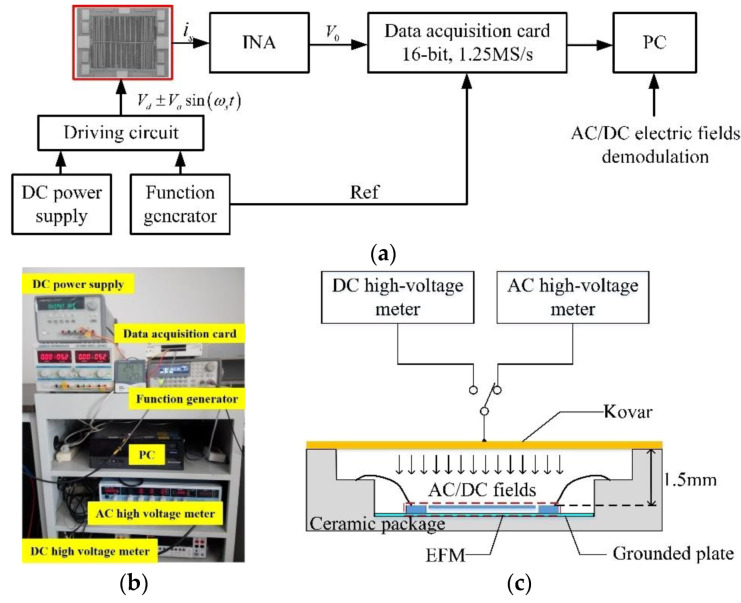
Verification test system for the AC/DC electric fields demodulation methods: (**a**) The schematic diagram; (**b**) The test setup; (**c**) A cross-section of the packaged EFM.

Figure 10a shows the schematic diagram of the verification test system for the AC/DC fields demodulation methods. First, the DC power supply and function generator were used to generate the driving voltages to excite the EFM. Second, the differential current outputs of the EFM would be converted into a voltage by the INA with a gain of 20 MΩ. Then, a 16-bit data acquisition (DAQ) card was used to collect both the output voltage and AC drive signals. Finally, the demodulation methods proposed in Section 3 were employed to extract the measured electric fields on a PC. The specific test setup is shown in Figure 10b. A DC high-voltage meter (Keithley 2410) with an accuracy of 0.02% and an AC high-voltage meter (Yokogawa 2558A) with an accuracy of 0.05% were used to generate DC electric fields and AC electric fields, respectively. In addition, the frequency range of the AC high-voltage meter was 40 Hz–1 kHz (0.001 Hz resolution).

The EFM was mounted in a ceramic package with a cover plate of Kovar, as shown in Figure 10c. The measured uniform and perpendicular electric fields were produced by applying DC or AC voltages to the cover plate of the package held 1.5 mm above the EFM. According to the simulation results of the literature [31], the electric fields perpendicular to the EFM electrodes inside the package are sufficiently uniform, and their values can be expressed with the voltage applied to the cover plate *V_c_* as:

(19) $$E_{n}=\frac{V_{c}}{1.5\times 10^{-3}m}$$

To avoid noise interference, the INA circuit was protected by a grounded metal case. The tests were demonstrated in ambient air at room temperature.

### 4.2. Testing and Analysis of Signal Characteristics

**Figure 11:**
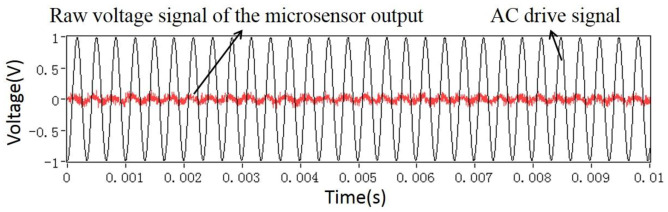
Raw voltage signal of the microsensor output and AC drive signal.

**Figure 12:**
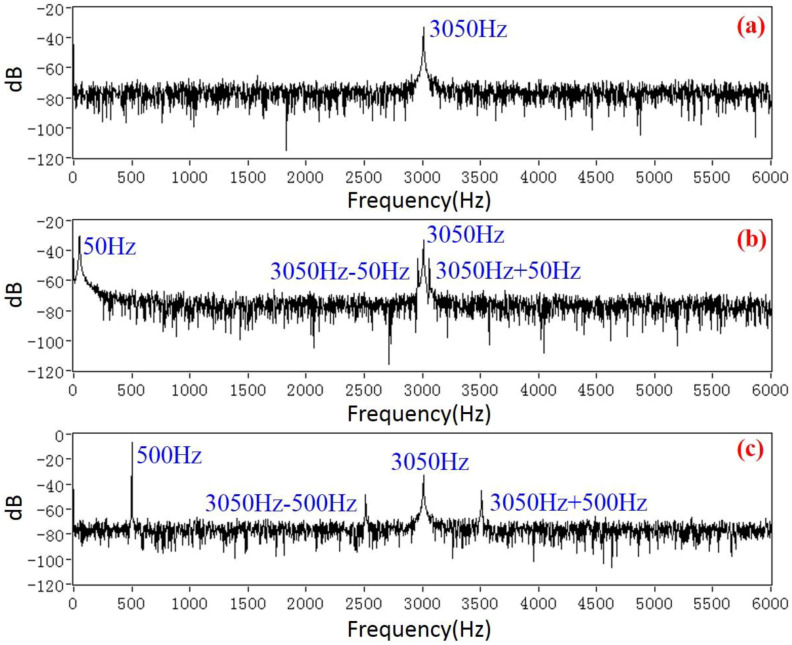
Spectrum of the microsensor output voltage: (**a**) under a DC electric field; (**b**) under a 50 Hz power frequency field; and (**c**) under a 500 Hz AC field.

**Figure 13:**
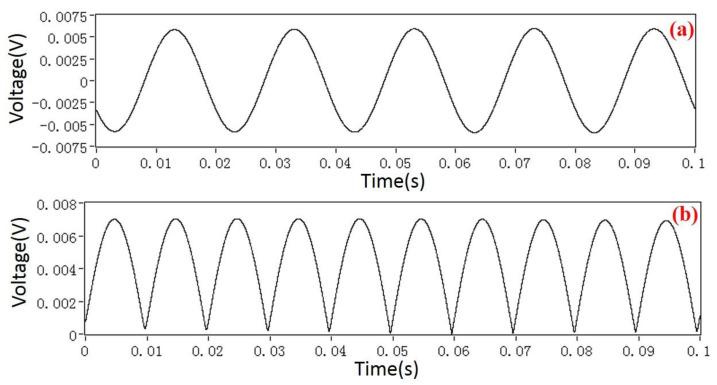
Demodulation results of a 50 Hz power frequency field using the HOCBM: (**a**) The output of *X*(*t*); (**b**) The output of *R*(*t*).

In order to gain a high sensitivity, the EFM was driven at a resonant frequency of 3050 Hz. Therefore, the frequency bandwidth of the AC/DC fields that the EFM could detect was [0, 1525 Hz). The characteristics and demodulation results of the EFM voltage output signals were displayed and analyzed by using LABVIEW software (version 2012). Figure 11 shows the raw voltage signal indicated by a red line from the microsensor, and the AC drive signal indicated by a black line. The spectrum of the sensor voltage output when a DC field is applied is shown in Figure 12a. The only frequency component is 3050 Hz, which is consistent with the result expressed by Equation (4). Furthermore, when a 50 Hz power frequency field is applied, the actual experiment reveals that there are four frequency components in the voltage signal of the microsensor output, which are 50 Hz, 3050 Hz − 50 Hz, 3050 Hz, and 3050 Hz + 50 Hz, respectively, as shown in Figure 12b. The actual experimental result has a frequency component of 3050 Hz more than the theoretical analysis result in Figure 5. The reason for this is that the AC drive-signal feedthrough of the microsensor is not considered during the theoretical analysis. Figure 12c shows the spectrum of the output voltage of the sensor applied under a 500 Hz AC field, which has the same characteristics as the 50 Hz power frequency field. Using the HOCBM, the *X*(*t*) and *R*(*t*) outputs obtained by demodulating a 50 Hz power frequency field are shown in Figure 13, which are in accordance with the normalized simulation results of Figure 7 and Figure 8, respectively.

### 4.3. Demodulation Results

**Figure 14:**
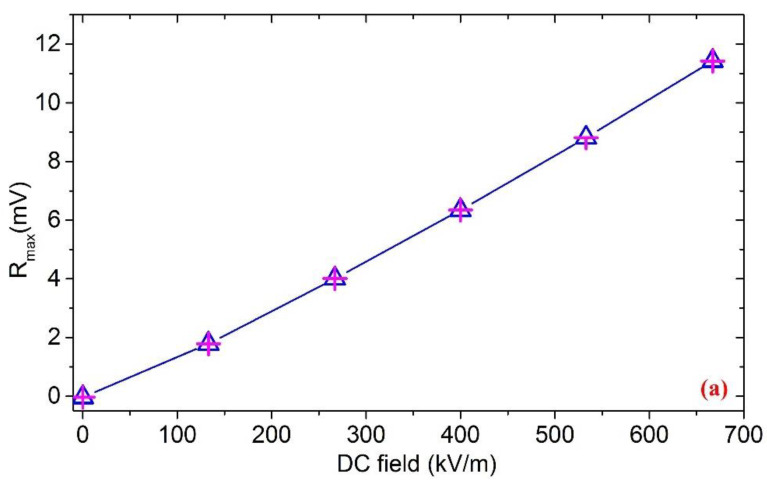

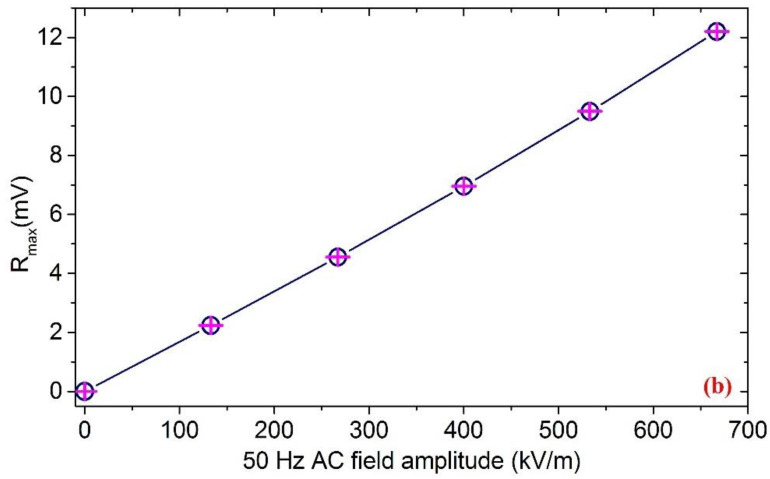
The response characteristics of the EFM: (**a**) The uncertainty of 2.4% for DC fields; (**b**) The uncertainty of 1.5% for 50 Hz power frequency fields.

**Figure 15:**
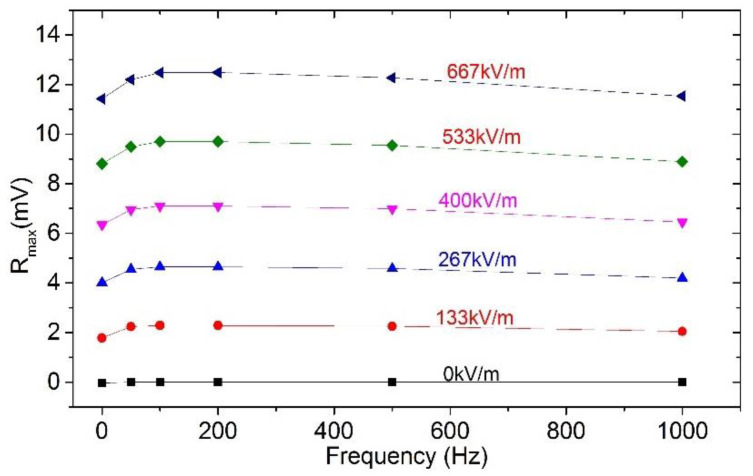
The frequency characteristics of the EFM.

Based on the HOCLBM, the response characteristics of the EFM for the most common DC and 50 Hz power frequency electric fields were tested within an electric field range of 0–667 kV/m, and the experimental results with error bars are shown in Figure 14. The detailed test results are listed in Table 1 and Table 2. The uncertainties of the three roundtrip measurements were calculated as 2.4% and 1.5% (see the Appendix A for the detailed calculation method of the uncertainty), respectively, which indicated that the microsensor has a high precision for the DC and 50 Hz power frequency field measurements. The frequency characteristics of the microsensor under different electric field amplitudes, obtained by experiments, are illustrated in Figure 15. The test results were in agreement with the ones delivered from the analysis in Section 3.3, indicating that within the frequency bandwidth of the detectable fields, the responses of the microsensor are identical for electric fields with the same amplitude and different frequencies. Nevertheless, there were some deviations in the actual test results. The detailed test data of a single trip are shown in Table 3. The test error increases with the magnitude of the measured electric field. Moreover, as the measured field frequency gradually increases, the demodulated output *R_max_* slightly decreases. The possible reasons for this include: (a) Since the distance between the cover plate of the package and the EFM is only 1.5 mm, the very small voltage change of the high-voltage meters will give rise to a relatively large electric field variation inside the package; (b) Using different types of high-voltage meters to generate DC fields and AC fields, respectively; and (c) Being affected by the filter order and the bandwidth of the INA.

## 5. Conclusions

In this work, the output signal characteristics and demodulation methods originating from the synchronous detection of the resonant EFMs are comprehensively studied, both analytically and experimentally. The synchronous demodulation, the HOCBM, and the HOCLBM are proposed for reconstructing the DC field, power frequency field, and AC/DC hybrid fields, respectively. The theoretical analysis shows that the frequency bandwidth of the detectable electric field for the EFM is [0, *ω_s_*/2). In addition, the sensitivity of the EFM for any frequency component of the AC/DC hybrid fields in the measurable frequency range is a fixed value |*k_q_X_r_R_f_ ω_s_*|. The experimental results demonstrated that within electric field ranges of 0–667 kV/m, the uncertainties were 2.4% and 1.5% for the most common DC and 50 Hz power frequency fields, respectively. The frequency characteristic test results of the microsensor were in agreement with the theoretical analysis in the range of 0–1 kHz under different electric field amplitudes. The proposed demodulation methods are mainly used to extract the measured DC field, power frequency field, or AC/DC hybrid fields from the output voltages of the microsensor, which is very helpful in promoting the application of the resonant EFMs in power systems.

## Figures and Tables

**Table 1:** The detailed test results of the response characteristics for DC fields.

Roundtrip	0 kV/m	133 kV/m	267 kV/m	400 kV/m	533 kV/m	667 kV/m
1st direct journey	−0.034 mV	1.785 mV	4.011 mV	6.344 mV	8.808 mV	11.432 mV
1st reverse journey	−0.035 mV	1.783 mV	4.006 mV	6.342 mV	8.810 mV	11.426 mV
2nd direct journey	−0.031 mV	1.785 mV	4.011 mV	6.346 mV	8.809 mV	11.426 mV
2nd reverse journey	−0.031 mV	1.788 mV	4.008 mV	6.344 mV	8.809 mV	11.422 mV
3rd direct journey	−0.035 mV	1.781 mV	4.010 mV	6.343 mV	8.809 mV	11.427 mV
3rd reverse journey	−0.036 mV	1.789 mV	4.010 mV	6.343 mV	8.810 mV	11.422 mV

**Table 2:** The detailed test results of the response characteristics for 50 Hz power frequency fields.

Roundtrip	0 kV/m	133 kV/m	267 kV/m	400 kV/m	533 kV/m	667 kV/m
1st direct journey	0.003 mV	2.233 mV	4.550 mV	6.952 mV	9.487 mV	12.200 mV
1st reverse journey	0 mV	2.236 mV	4.550 mV	6.954 mV	9.494 mV	12.196 mV
2nd direct journey	0.001 mV	2.236 mV	4.543 mV	6.955 mV	9.495 mV	12.202 mV
2nd reverse journey	0.002 mV	2.236 mV	4.553 mV	6.953 mV	9.488 mV	12.198 mV
3rd direct journey	0.001 mV	2.238 mV	4.550 mV	6.954 mV	9.494 mV	12.204 mV
3rd reverse journey	0.001 mV	2.241 mV	4.551 mV	6.952 mV	9.492 mV	12.199 mV

**Table 3:** The detailed test data of the EFM frequency characteristics under different electric fields.

Frequency (Hz)	0 kV/m	133 kV/m	267 kV/m	400 kV/m	533 kV/m	667 kV/m
0	−0.03 mV	1.79 mV	4.01 mV	6.34 mV	8.81 mV	11.43 mV
50	0	2.24 mV	4.55 mV	6.95 mV	9.49 mV	12.20 mV
100	0	2.28 mV	4.65 mV	7.10 mV	9.70 mV	12.48 mV
200	0	2.27 mV	4.65 mV	7.10 mV	9.70 mV	12.49 mV
500	0	2.25 mV	4.58 mV	6.99 mV	9.54 mV	12.27 mV
1000	0	2.05 mV	4.19 mV	6.46 mV	8.89 mV	11.53 mV
Absolute error	0.03 mV	0.5 mV	0.63 mV	0.75 mV	0.89 mV	1.06 mV

## Appendix A

The uncertainty refers to a limit range where the deviation of the actual characteristics of the microsensor from its reference operating characteristics does not exceed under the reference operating conditions [36]. The smaller the uncertainty, the higher the credibility of the measurement result.

Take *m* (normally, *m* = 5–11) calibration points (including the upper-lower boundaries) uniformly within the measurement range of the electric field. First, starting from the lower boundary of the measurement range, the electric field is steadily applied according to the specified calibration point. After the stability, the output value of the microsensor is read. Second, perform the same operation at the remaining calibration points in order until the upper boundary of the measurement range. Finally, the upper boundary of the electric field will fluctuate upward by about 0.2% and then return to the upper boundary; the output value read at this time is used as the initial value of the reverse journey, and the measurement is performed in the reverse order of the original calibration points. Both the direct journey and reverse journey are executed *n* times (*n* = 3).

The Bessel formula is used to calculate the standard deviation of the direct journey subsamples and the reverse journey subsamples at each calibration point, respectively.

The standard deviation *S_Ui_* of the i-th calibration point subsamples of the direct journey is calculated by:

(A1) $$S_{Ui}=\sqrt{\frac{1}{n-1}\sum_{j=1}^{n}{\left(Y_{Uij}-{\bar{Y}}_{Ui}\right)}^{2}}\left(i=1,2\cdots m;j=1,2\cdots n\right)$$

where *Y_Uij_* is the j-th measurement value of the i-th calibration point of the direct journey, and ${\bar{Y}}_{Ui}$ is the average value of the direct journey measurement at the i-th calibration point.

The standard deviation *S_Di_* of the i-th calibration point subsamples of the reverse journey is calculated by:

(A2) $$S_{Di}=\sqrt{\frac{1}{n-1}\sum_{j=1}^{n}{\left(Y_{Dij}-{\bar{Y}}_{Di}\right)}^{2}}\left(i=1,2\cdots m;j=1,2\cdots n\right)$$

where *Y_Dij_* is the j-th measurement value of the i-th calibration point of the reverse journey, and ${\bar{Y}}_{Di}$ is the average value of the reverse journey measurement at the i-th calibration point.

Thus, the sub-sample standard deviation *S* of the microsensor in the entire measurement range is given by:

(A3) $$S=\sqrt{\frac{1}{2m}\left(\sum_{i=1}^{m}S_{Ui}^{2}+\sum_{i=1}^{m}S_{Di}^{2}\right)}$$

Additionally, both the system error ∆*Y_LH,Ui_* of the direct journey and the system error ∆*Y_LH,Di_* of the reverse journey are, relative to the reference straight line, calculated by:

(A4) $$\begin{array}{l} \Delta Y_{LH,Ui}={\bar{Y}}_{Ui}-Y_{i}\\ \Delta Y_{LH,Di}={\bar{Y}}_{Di}-Y_{i} \end{array}\left(i=1,2\cdots m\right)$$

where *Y_i_* is the value of the reference straight line of the microsensor at the i-th calibration point.

The uncertainty of the direct journey *ξ_LHR,Ui_* and the uncertainty of the reverse journey *ξ_LHR,Di_* are given by:

(A5) $$\begin{array}{l} \xi_{LHR,Ui}=\frac{\left|\Delta Y_{LH,Ui}+2S\right|}{Y_{FS}}\times 100\%\\ \xi_{LHR,Di}=\frac{\left|\Delta Y_{LH,Di}+2S\right|}{Y_{FS}}\times 100\% \end{array}$$

where *Y_FS_* is the full-scale output of the microsensor.

Therefore, the uncertainty of the roundtrip measurements *ξ_LHR_* can be expressed as:

(A6) $$\xi_{LHR}=\max\left|\xi_{LHR,Ui},\xi_{LHR,Di}\right|$$
